# Supplementary material for: Lacticaseibacillus rhamnosus FM9 and Limosilactobacillus fermentum Y57 Are as Effective as Statins at Improving Blood Lipid Profile in High Cholesterol, High-Fat Diet Model in Male Wistar Rats
Source: Nutrients. 2022 Apr 15;14(8):1654. doi: 10.3390/nu14081654 (PMC9027066; doi:10.3390/nu14081654)
Supplement: Supplementary file 1 [file nutrients-14-01654-s001.zip › nutrients-1615110-supplementary.pdf]

Supplementary Data

Figure S1 (a–h): GIT related stress of LAB isolates

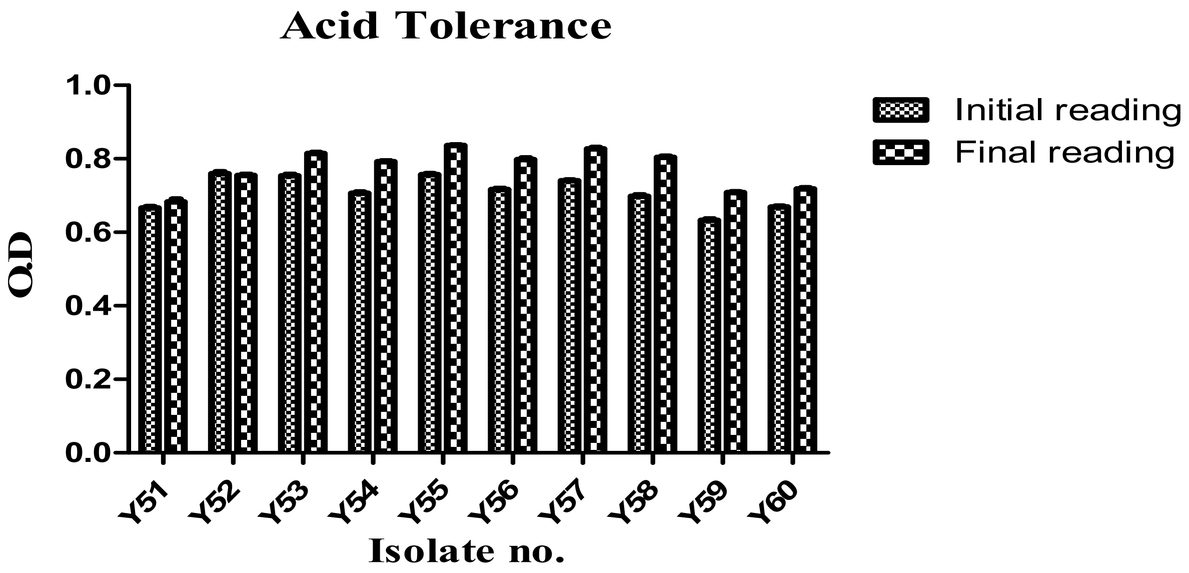

Figure S1(a): comparative analysis of phenol tolerance of isolates

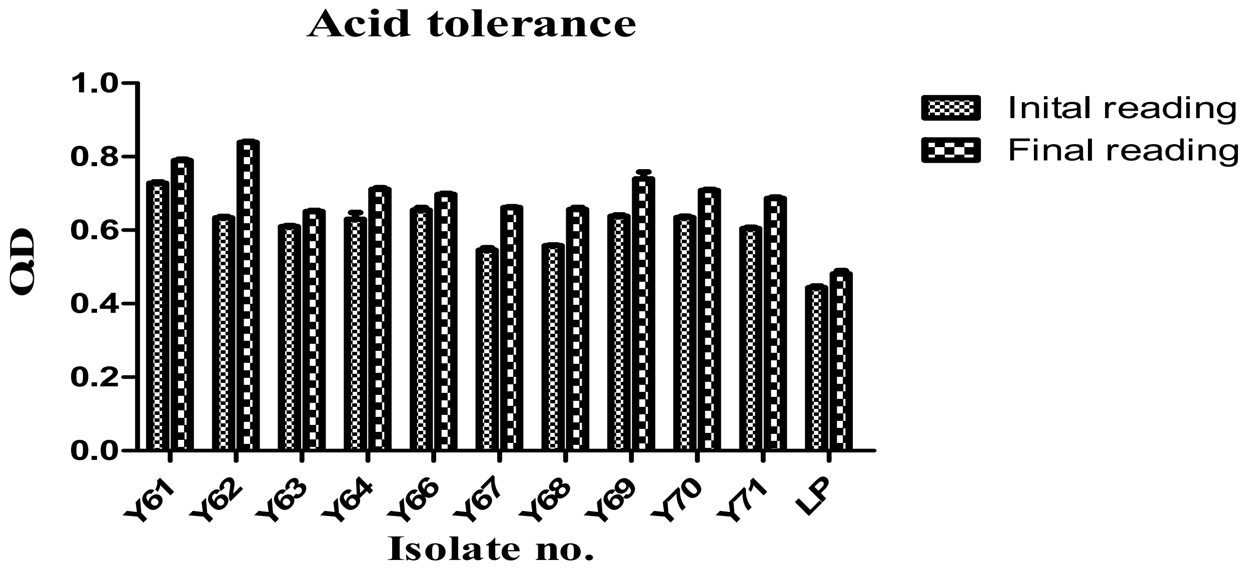

Figure S1(b): comparative analysis of phenol tolerance of isolate

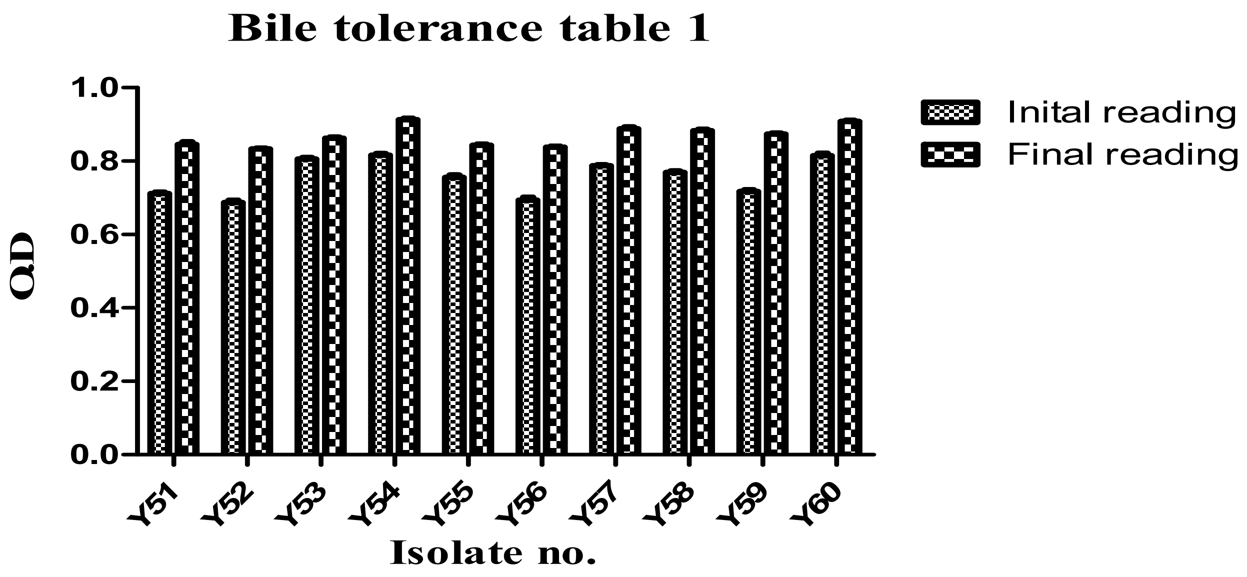

Figure S1(c): comparative analysis of phenol tolerance of isolates

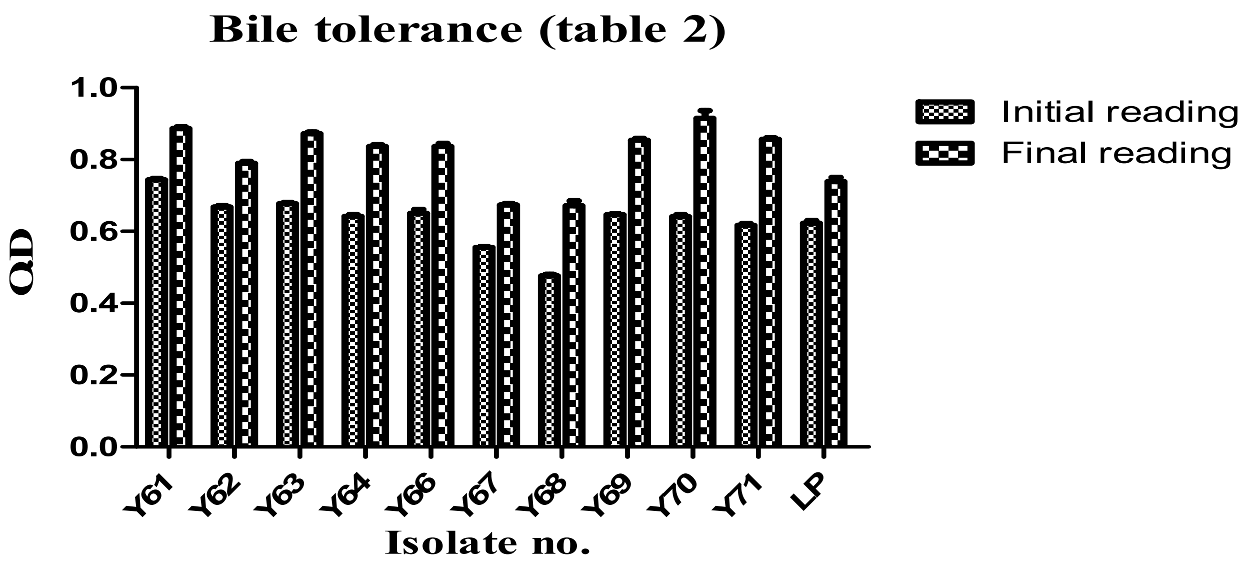

Figure S1(d): comparative analysis of bile tolerance of isolates

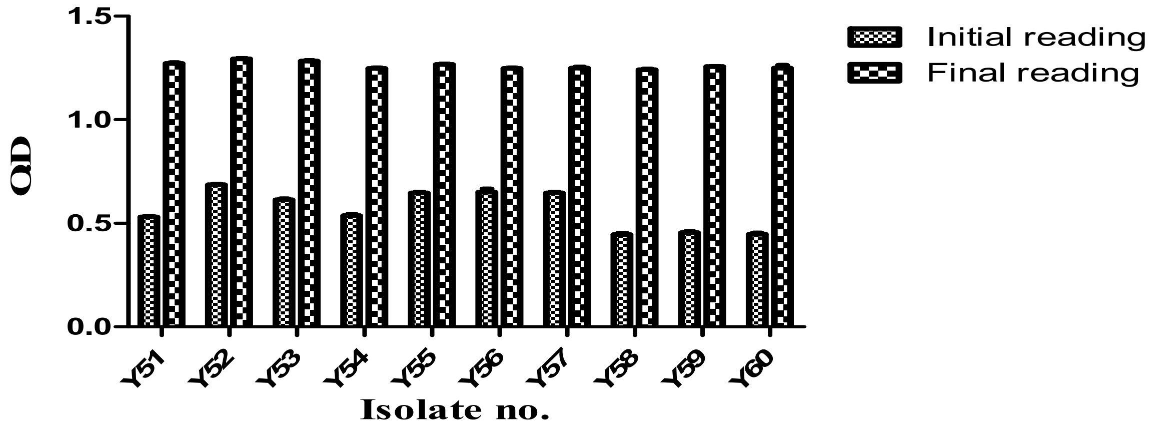

Figure S1(e): comparative analysis of phenol tolerance of isolates

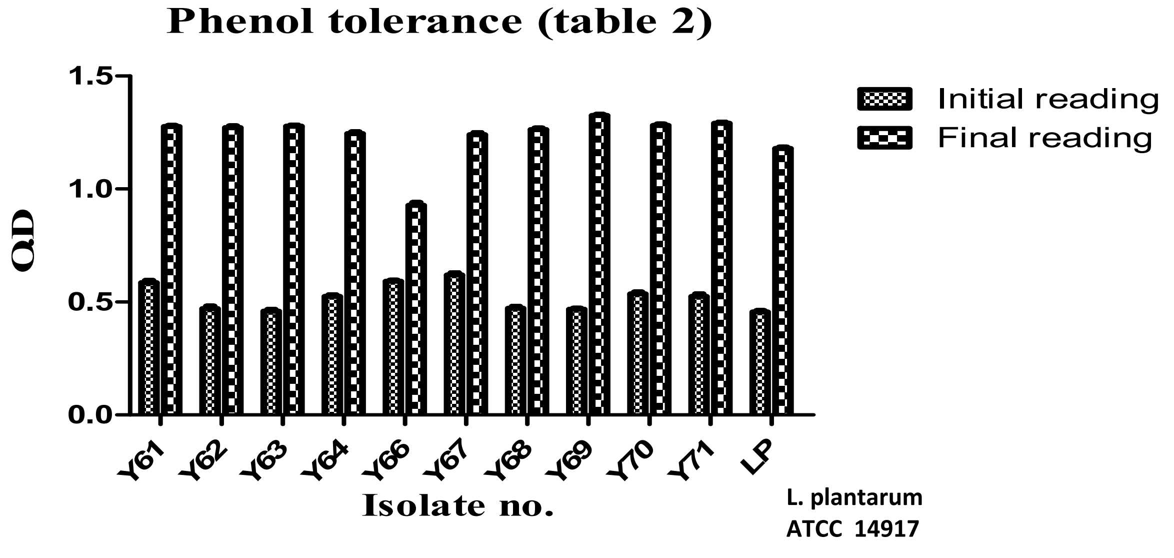

Figure S1(f): comparative analysis of phenol tolerance of isolates

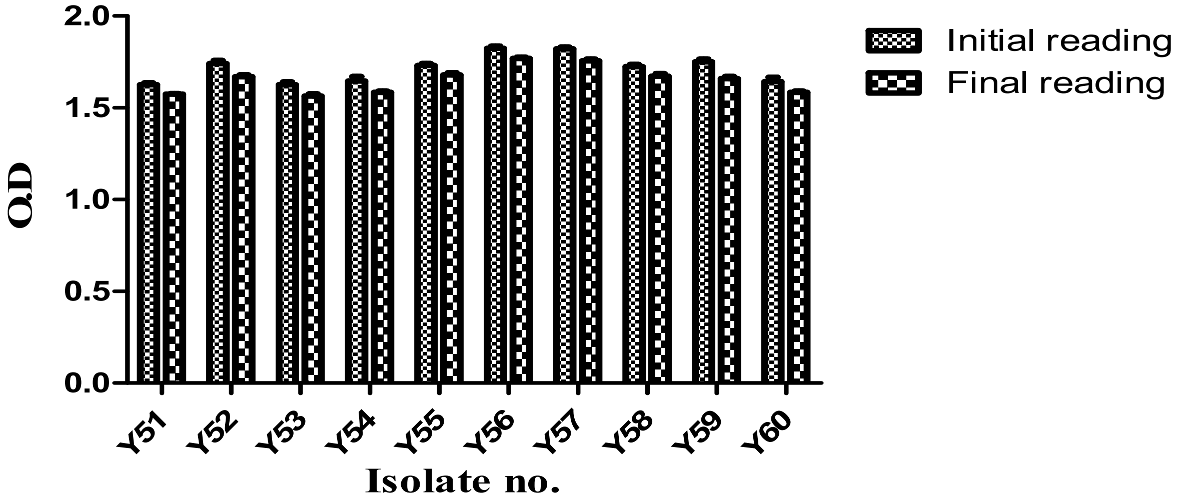

Figure S1(g): comparative analysis of lysozyme tolerance of isolate

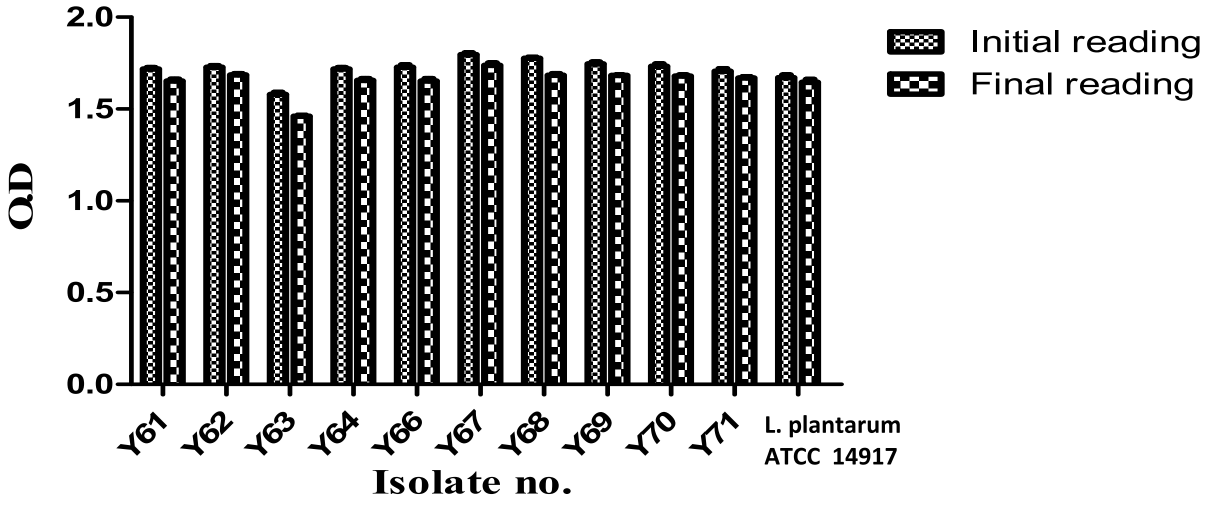

Figure S1(h): comparative analysis of lysozyme tolerance of isolates
